# Deep enrichment of soil *Proteobacteria* and its coupled response to carbon, nitrogen, and phosphorus cycles under quizalofop-p-ethyl stress

**DOI:** 10.3389/fmicb.2026.1766973

**Published:** 2026-03-12

**Authors:** Huan Meng, Yuanlong Chen, Lili Yang, Yueyao Li, Zhiling Nie, Jiayi Cao, Jianan Du, Chuanzhen Ma, Yushuai Wei, Fengshan Yang, Haiyan Fu

**Affiliations:** Engineering Research Center of Agricultural Microbiology Technology, Ministry of Education & Heilongjiang Provincial Key Laboratory of Ecological Restoration and Resource Utilization for Cold Region & Key Laboratory of Molecular Biology, College of Heilongjiang Province & School of Life Sciences, Heilongjiang University, Harbin, China

**Keywords:** carbon, nitrogen, and phosphorus cycles, functional regulation, herbicide degradation, *Proteobacteria*, quizalofop-p-ethyl, soil depth

## Abstract

To investigate the vertical impacts of quizalofop-p-ethyl stress on soil bacterial communities and their ecological functional responses in wheat fields, this study collected soil samples from three depths (0–30 cm, 30–60 cm, and 60–90 cm) using a grid sampling method in typical wheat fields of Inner Mongolia Autonomous Region. Through quizalofop-p-ethyl acclimation experiments with concentration gradients (50–300 mg/L), combined with bacterial community structure and functional analyses, this study focused on revealing the dominant enrichment of *Proteobacteria* in deep soil and its key regulatory role in carbon (C), nitrogen (N), and phosphorus (P) cycles. The results showed that quizalofop-p-ethyl treatment significantly altered soil microbial community structure and induced obvious functional remodeling. As a core responsive taxon, the relative abundance of *Proteobacteria* increased significantly with increasing soil depth, becoming the absolute dominant phylum in deep soil layers. This change was significantly positively correlated with the upregulation of key metabolic pathways involved in soil C, N, and P cycles (including the citrate cycle, nitrogen metabolism, phosphonate metabolism, etc.). Functional gene analysis further indicated that the expression of multiple genes related to nitrogen assimilation and phosphorus utilization was closely associated with the abundance of *Proteobacteria*, directly promoting N and P cycling processes. Meanwhile, the activation of quizalofop-p-ethyl degradation-related pathways provided additional carbon sources for microorganisms, synergistically enhancing the C cycle. From the perspective of “dominant bacterial taxa driving element cycling,” this study clarified the vertical differentiation mechanism of soil microbial ecological functions under quizalofop-p-ethyl stress, which deepens the understanding of the soil microecological effects of herbicides.

## Graphical abstract

**Figure:**
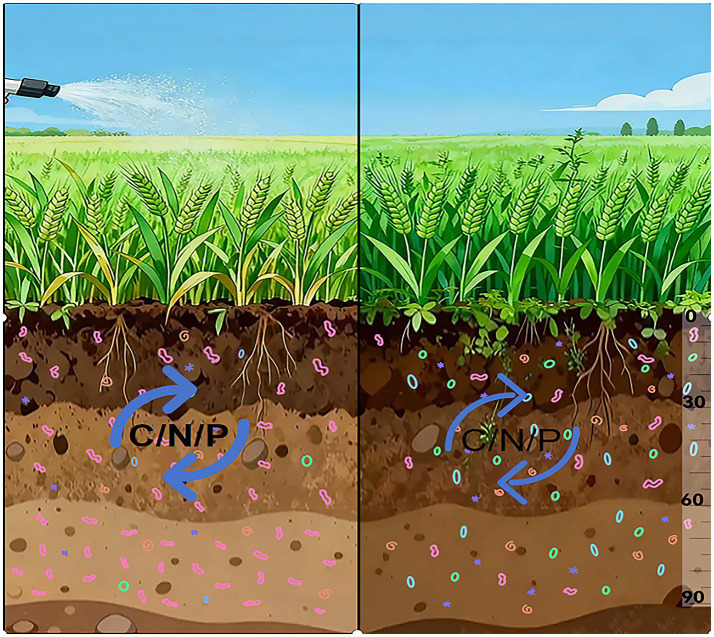

## 1 Introduction

Soil microorganisms are an important component of agricultural ecosystems (Jia et al., 2025), and their responses to agricultural management practices (such as herbicide application) are directly related to soil health and the stability of ecological functions. Quizalofop-p-ethyl is a selective herbicide specifically targeting gramineous weeds. It exhibits high safety to local major food crops such as wheat and corn, while demonstrating remarkable efficacy in controlling target weeds. Therefore, it has become a commonly used herbicide in local food crop fields including wheat fields, and is widely applied in agricultural production to ensure crop yields. It not only inhibits target weeds but also exerts stress on non-target microbial communities after entering the soil. Quizalofop-p-ethyl can induce the glutathione conjugation detoxification mechanism mediated by the PfGSTF2 gene in plants (e.g., the development of resistance to quizalofop-p-ethyl in *Polypogon fugax*) and affect crop yield components in combination with safeners (Chen et al., 2025); meanwhile, microorganisms such as *Methylobacterium populi* YC-XJ1 can achieve its cometabolic degradation through its unique esterase QPHE1, demonstrating the key role of microorganisms in herbicide transformation (Li et al., 2020).

Soil bacteria play a crucial role in maintaining soil fertility and ecosystem functions, particularly in driving the biogeochemical cycles of key elements such as carbon, nitrogen, and phosphorus (Osburn et al., 2024; Liu et al., 2025; Peng et al., 2026; Zang et al., 2026). Herbicide application significantly affects soil microbial community structure and nitrogen cycling functions (He et al., 2024; Zhang Y. et al., 2025b), alters the structure and complexity of rhizosphere microbial networks, and thereby influences nitrogen transformation processes (Ma et al., 2022). The application of monosulfuron inhibits key enzyme activities, reduces nutrient transformation, and damages functional microbial communities, leading to decreased soil nutrient supply capacity, weakened pollutant degradation ability, and degradation of ecological service functions (Feng et al., 2025).

The structure and function of soil microbial communities are not homogeneously distributed but exhibit obvious vertical differentiation patterns with soil depth (Zhu et al., 2024; Hui et al., 2025; Zhang Z. et al., 2025b). Compared with topsoil, the microbial communities and their functions in deep agricultural soils generally show weaker resistance to global changes, reflecting the functional differentiation of microorganisms at different depths in responding to environmental stress (Peng et al., 2025). Driven by carbon availability, deep-soil microorganisms display distinct response patterns of nitrogen use efficiency, and the accumulation of their extracellular polymers is closely associated with the deep sequestration of soil organic carbon (Zhang Q. et al., 2025). Functional differentiation of microbial communities along the depth gradient is one of the important factors leading to vertical differences in element cycles (Dupont et al., 2014; Yuan et al., 2018; Qian et al., 2023). However, current research on how herbicide stress affects the vertical pattern of microbial functions in soil profiles and further regulates carbon, nitrogen, and phosphorus cycling processes remains relatively limited.

Based on the above background, this study took typical wheat field soils as the research object. By simulating a quizalofop-p-ethyl stress environment, it systematically analyzed the changes in bacterial community structure in soil layers of different depths and their coupled relationships with the expression of functional genes involved in carbon, nitrogen, and phosphorus cycles. Three key scientific questions were focused on: (1) Whether there are concentration- and depth-dependent response thresholds of microbial communities to quizalofop-p-ethyl stress; (2) Whether and how quizalofop-p-ethyl application affects the vertical differentiation pattern of soil bacterial communities; (3) How changes in soil microbial structure further influence the depth-specific characteristics of soil carbon, nitrogen, and phosphorus cycles. The results of this study aim to provide a theoretical basis for in-depth understanding of the impact mechanism of herbicides on vertical ecological processes of soil microorganisms, as well as microbial reference for the safety and sustainable management of agricultural ecosystems.

## 2 Materials and methods

### 2.1 Soil sampling and processing

**Figure 1:**
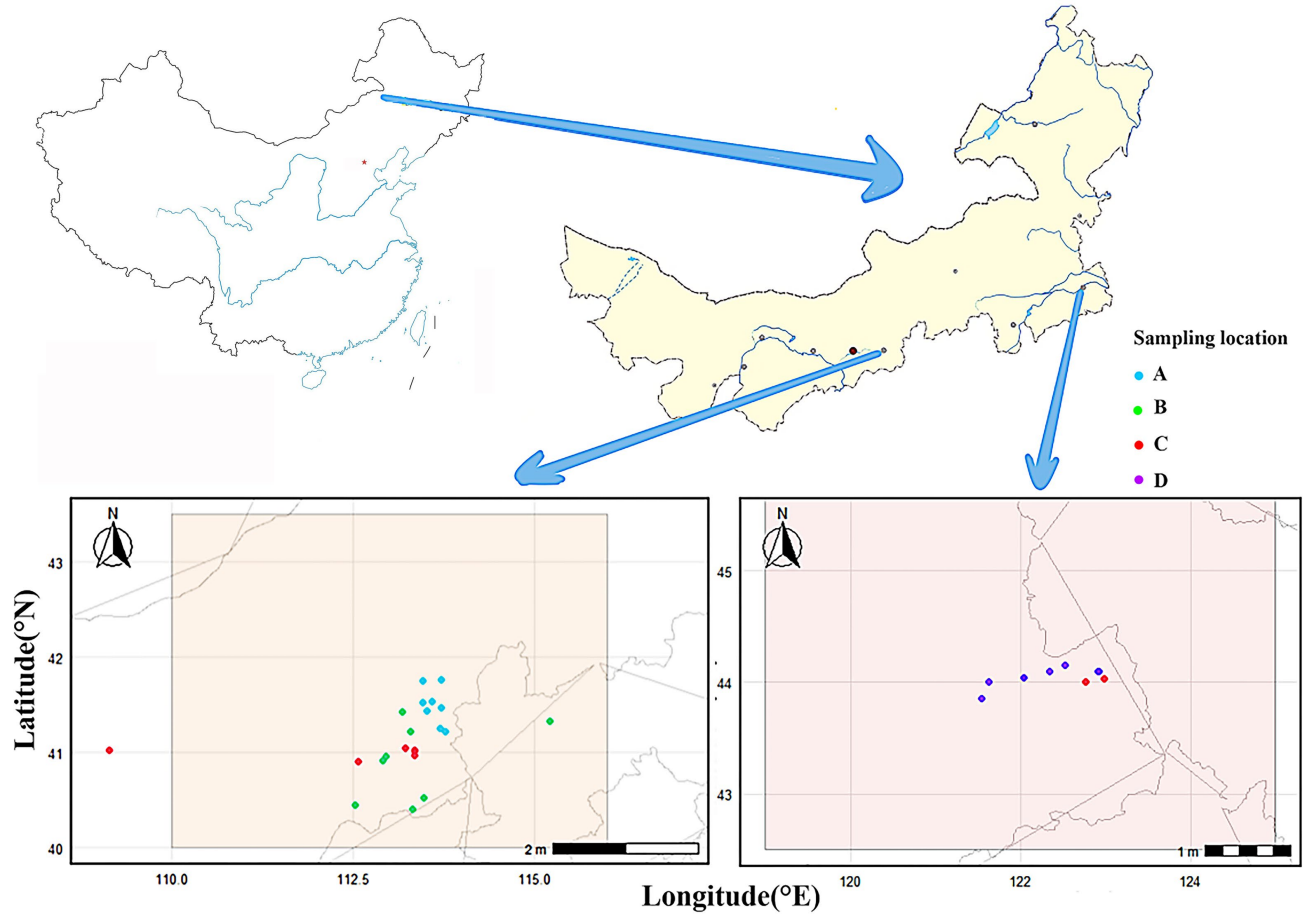
Map of village information for soil sampling plots. A total of 32 wheat field plots were distributed in Wulanchabu City (Plot Nos. A1–A8, B1–B8, C1–C6) and Tongliao City (Plot Nos. C7, C8, D1–D8).

Soil samples were collected from 32 wheat field plots in Wulanchabu City (plots A1–A8, B1–B8, C1–C6) and Tongliao City (plots C7–C8, D1–D8) of Inner Mongolia Autonomous Region (Figure 1 and Supplementary Table 1). All plots had consistent planting history (continuous wheat cultivation for ≥5 years) and no prior application of quizalofop-p-ethyl to avoid interference from historical herbicide residues. During the wheat tillering stage in May 2023, a 10 m × 10 m grid-based five-point sampling method was adopted for each plot: five sampling points were evenly arranged, and soil cores at three depths (0–30 cm, 30–60 cm, and 60–90 cm) were collected using a soil auger with an inner diameter of 5 cm. Soil samples from the same depth in the same plot were thoroughly mixed to prepare composite samples weighing ≥1 kg. All composite samples were placed in sterile polyethylene bags, transported back to the laboratory within 4 h under ice bath conditions, stored at 4 °C, and pretreated within 24 h to minimize changes in microbial activity. Total soil nitrogen content was determined by the semi-micro Kjeldahl method; available phosphorus content was extracted with 0.5 mol/L NaHCO₃ and then measured via the molybdenum-antimony anti-colorimetric method; available potassium content was extracted with 1 mol/L ammonium acetate and determined using the flame photometric method. All indicators were assayed with three technical replicates.

### 2.2 Quizalofop-p-ethyl treatment and bacterial growth determination

Experimental materials included quizalofop-p-ethyl (purity ≥98%, Hubei Bolan Chemical Co., Ltd.), analytical grade acetone (Sinopharm Group Co., Ltd.), sterile mineral salt medium (1.79 g/L K₂HPO₄, 0.45 g/L KH₂PO₄, 0.2 g/L MgSO₄·7H₂O, 0.4 g/L NaCl, pH 7.0), LB medium, and 0.85% normal saline. Instruments used included an autoclave, shaking incubator, high-speed refrigerated centrifuge, microplate reader, etc. (Supplementary Tables 2, 3). Soil samples were air-dried at room temperature to a moisture content of 10–12%, ground aseptically, and passed through a 1 mm sieve. A 0.15 g aliquot of sieved soil was mixed with 1.2 mL of sterile normal saline by vortexing, then allowed to stand at 4 °C for 2 h; the supernatant was collected as bacterial suspension. Quizalofop-p-ethyl was dissolved in acetone to prepare a 50 mg/mL stock solution, which was filtered through a 0.45 μm organic filter membrane for sterilization and then diluted with sterile mineral salt medium to gradient concentrations of 50, 100, 150, 200, 250, and 300 mg/L (Supplementary Table 4). A solvent control (medium containing an equal volume of acetone) and a blank control (sterile mineral salt medium only) were set up. Acclimatization experiments were conducted in 96-well deep-well plates with 3 replicates per treatment. A 200 μL aliquot of bacterial suspension was added to 1,200 μL of gradient herbicide solutions, followed by incubation in a shaking incubator at 30 °C and 180 rpm for 6 days. The OD₆₀₀ values before (initial A₀) and after acclimatization (A₁) were measured using a microplate reader. Bacterial growth rate (%) was calculated by the formula: [(A₁ − A₀)/A₀] × 100. The 6 plots with the highest bacterial growth rates were selected, and a total of 18 *in-situ* soil samples (3 depths per plot) were collected for subsequent microbial community analyses. Strict sterilization was performed throughout the experiment to control contamination and ensure experimental reproducibility.

### 2.3 DNA extraction and 16S rRNA sequencing

DNA was extracted using the PowerSoil^®^ DNA Isolation Kit. Briefly, 250 μL of the sample and 60 μL of Solution C1 were added to PowerBead Tubes. After vortexing at 3,200 rpm for 10 min, the tubes were centrifuged at 10,000 × g for 30 s. Solution C2 was sequentially added to the supernatant, followed by incubation at 4 °C for 5 min and centrifugation; the same incubation and centrifugation steps were repeated after adding Solution C3. Subsequently, 1,200 μL of Solution C4 was added to the supernatant, which was then passed through a Spin Filter. The filter was washed with Solution C5 and DNA was eluted with Solution C6. Qualified DNA samples were verified by 1% agarose gel electrophoresis and Nanodrop 2000 detection, then stored at −20 °C for further use. The DNA was diluted to 15 ng/μL, and the V3–V4 hypervariable region was amplified in a 25 μL reaction system using the 338F/806R primer pair with 8 bp barcodes on an ABI 9700 PCR instrument. The PCR program was as follows: initial denaturation at 94 °C for 6 min; 35 cycles of annealing at 56 °C for 1 min and extension at 72 °C for 1 min; final extension at 72 °C for 10 min. PCR products were purified using Agencourt AMPure XP magnetic beads and detected with a Caliper LabChip GX Touch HT. Library construction was performed using the NEB Next kit: PCR products were mixed in proportion, target fragments were excised from a 2% agarose gel, quantified with a Qubit 2.0 Fluorometer, and subjected to end repair, adapter ligation, and PCR enrichment followed by magnetic bead purification. The final libraries were qualified by detection with Nanodrop 2000, Agilent 2100, and ABI Step One Plus, and sequenced on the Illumina Novaseq 6000 platform with PE250 mode.

### 2.4 Bioinformatics analysis

Raw sequencing data were filtered and assembled using Pear software (v0.9.6). Sequences containing ambiguous bases (N) or with a quality score below 20 were removed. During assembly, a minimum overlap length of 10 bp and a *p*-value threshold of 0.0001 were set. After assembly, sequences shorter than 230 bp were excluded using Vsearch software (v2.7.1), and chimeric sequences were eliminated by the Uchime algorithm combined with the Gold Database. Valid tags were clustered into operational taxonomic units (OTUs) at a 97% sequence similarity threshold using Uparse, and the most frequently occurring sequence in each OTU was selected as the representative sequence. Representative sequences of OTUs were aligned against the Silva 138 database via BLAST with an *e*-value cutoff of 1 × 10^−5^, and taxonomic annotation was performed using tools including BLAST and RDP Classifier.

### 2.5 Statistical analysis

All statistical graphs (including boxplots, PCoA plots, stacked bar charts, chord diagrams, volcano plots, forest plots, etc.) were generated in R using R packages such as dplyr, ggplot2, tidyr, openxlsx, WGCNA, vegan, and ape. After standardization of OTU data, OTUs with a total abundance below 0.01% across samples were excluded based on dual criteria of abundance and frequency, and OTUs with high abundance and high prevalence were retained for subsequent analyses (Dickie, 2010). Data analysis was performed using SPSS 20.0 software (IBM, Chicago, United States). Differences between two groups were evaluated by Student’s *t*-test, while differences among multiple groups were analyzed using one-way analysis of variance (ANOVA) followed by Tukey’s honest significant difference (HSD) test. Statistical significance of different treatments was indicated by *p*-values (^*^p < 0.05, **p < 0.01, and ^***^p < 0.001).

## 3 Results

### 3.1 Effects of gradient acclimation with quizalofop-p-ethyl on the growth amount of soil bacteria in wheat fields

**Figure 2:**
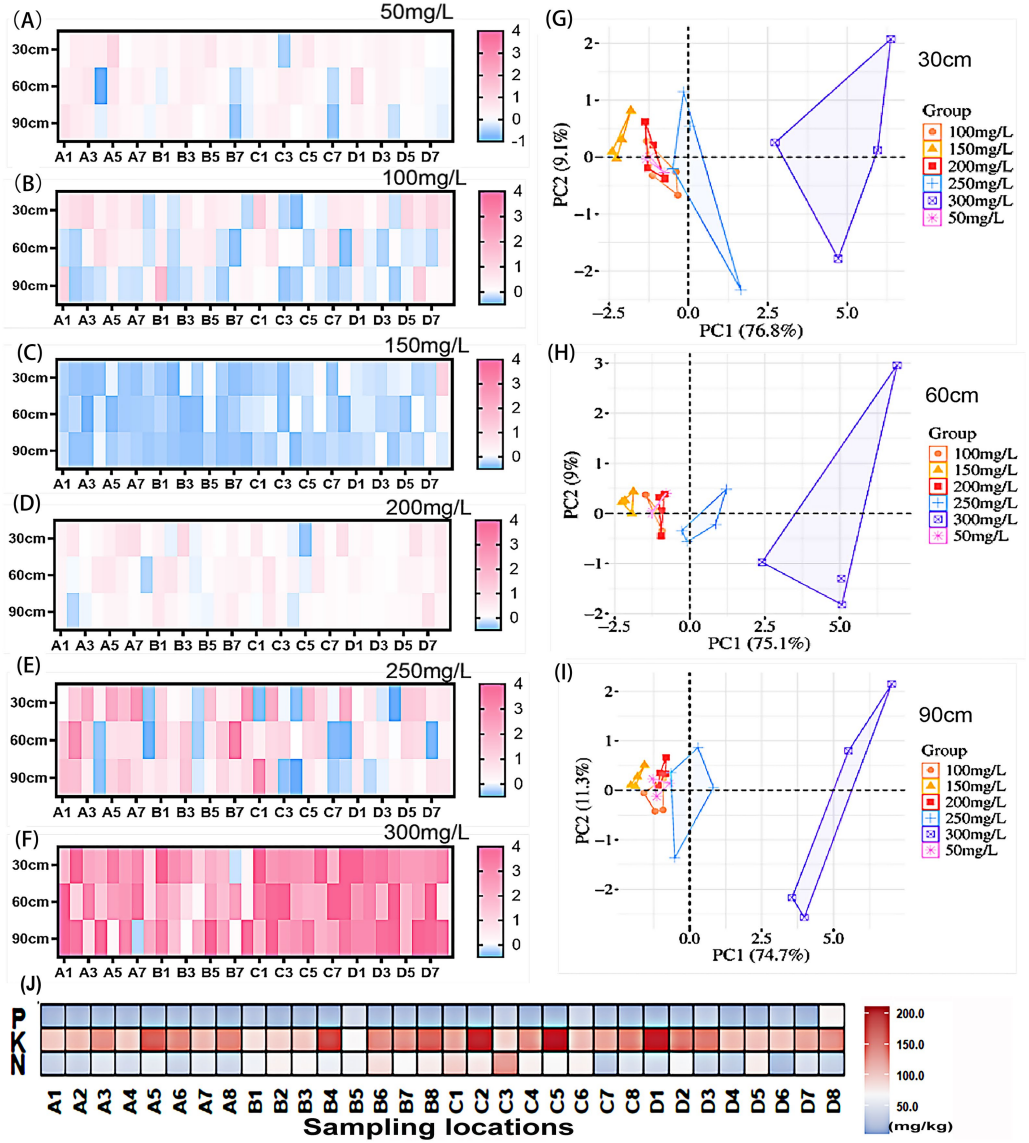
Effects of different concentrations of quizalofop-p-ethyl on soil bacterial biomass at different depths in wheat fields: **(A–F)** respectively show the response changes of bacterial growth rate in three soil depths (0–30 cm, 30–60 cm, 60–90 cm) under different concentrations of quizalofop-p-ethyl (50–300 mg L^−1^), with the horizontal axis representing 32 sampling locations and the vertical axis representing the three soil depths, where the shade of color indicates the magnitude of positive and negative changes in bacterial growth rate; **(G–I)** are the principal component analysis (PCA) plots of soils at the three depths (0–30 cm, 30–60 cm, 60–90 cm) after treatment with different concentrations of quizalofop-p-ethyl (50–300 mg L^−1^); **(J)** is a heatmap of nitrogen, phosphorus and potassium contents in in-situ field soils, with significant difference analysis conducted for the same indicator among different sampling locations, and no significant differences were observed.

Under low concentrations of quizalofop-p-ethyl (50–150 mg/L) (Figures 2A–C), the growth amount of soil bacteria exhibited significant concentration- and depth-dependent response differences: at 50 mg/L, the bacterial growth amount in most plots fluctuated between −0.5 and +1.0%, with obvious response differentiation among different depths; at 100 mg/L, the number of samples with inhibited bacterial growth was significantly higher than that at 50 mg/L; at 150 mg/L, bacterial growth was generally inhibited, and the fluctuation range in the 60–90 cm deep soil was larger than that in the topsoil and middle soil layers. Under high concentrations of quizalofop-p-ethyl (200–300 mg/L) (Figures 2D–F), the fluctuation range of bacterial growth amount was significantly expanded compared with medium and low concentrations, and the overall growth was positive: at 200 mg/L, the growth amount at different depths was still concentrated between −0.5 and +1.0%, but the proportion of samples with positive growth was higher than that at 150 mg/L; at 250 mg/L, the growth amount of specific depths (e.g., 60–90 cm) in some plots increased by up to 3%; at 300 mg/L, the fluctuation range expanded to 6%, mainly showing positive growth, indicating that high concentrations had induced the dominant growth of herbicide-resistant bacteria. Principal component analysis (PCA) results (Figures 2G–I) further revealed that the response of soil bacterial communities to quizalofop-p-ethyl was significantly depth-dependent. The community structure of the high-concentration (200–300 mg/L) treatment group was significantly separated and deviated remarkably with increasing depth. It is inferred that deep soil was most affected by the bacterial community, and resistant bacterial groups became dominant under high concentrations. Analysis of variance (ANOVA) confirmed (Figure 2J) that there were no significant differences in nutrient indicators among the sampling locations (*p* > 0.05). This result ensured the consistency of the baseline for subsequent microbial response analysis and eliminated the interference of inherent soil nutrient heterogeneity on the experimental outcomes.

Based on the above results, soil samples from plots B3, C1, C8, D2, D5, and D6 (designated as 1D, 2D, 3D, 4D, 5D, and 6D) under the 50 mg/L treatment were selected for subsequent sequencing. As the “initial response threshold” of quizalofop-p-ethyl stress, the 50 mg/L concentration can reflect the early regulatory effects of concentration and depth on bacterial growth, accurately capture the initial response characteristics of bacteria to pesticide stress, and avoid response deviations caused by the dominance of resistant bacterial groups under high concentrations. This concentration enables the authentic reflection of the interactive relationship between concentration, depth, and bacterial growth.

### 3.2 Variation trends of soil bacterial community structure at different depths induced by quizalofop-p-ethyl

**Figure 3:**
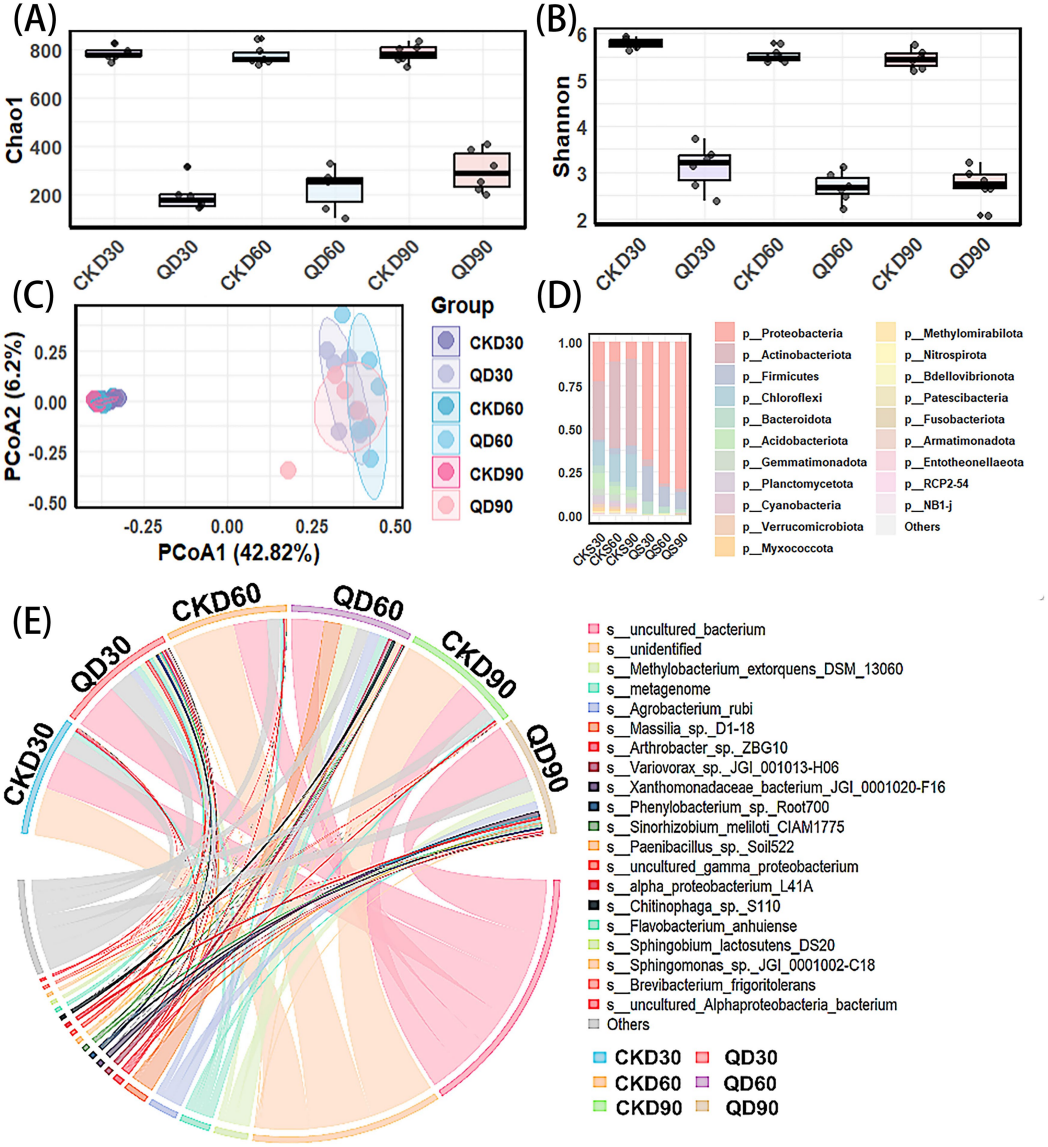
Variation trends of soil bacterial community structure at different depths induced by quizalofop-p-ethyl: **(A)** Boxplot of Chao1 index of soil bacterial communities in different treatment groups; **(B)** boxplot of Shannon index; **(C)** principal coordinate analysis (PCoA) plot based on Bray–Curtis distance; **(D)** stacked bar chart of microbial relative abundance at the phylum level; **(E)** chord diagram of species-treatment group associations at the species level. A total of six groups were set in the experiment, among which the control groups (before quizalofop-p-ethyl treatment) were CKD30, CKD60, and CKD90, and the treatment groups (after quizalofop-p-ethyl treatment) were QD30, QD60, and QD90, corresponding to three soil depths of 0–30 cm, 30–60 cm, and 60–90 cm, respectively.

Both the Chao1 index (Figure 3A) and Shannon index (Figure 3B) collectively revealed the significant effects of quizalofop-p-ethyl application on the α-diversity of soil bacteria in wheat fields. The application of quizalofop-p-ethyl significantly reduced the species richness and community evenness of soil bacteria, and this decreasing trend became more pronounced in the treatment groups with increasing soil depth. Principal coordinate analysis (PCoA) (Figure 3C) further demonstrated the differentiation characteristics of soil bacterial community structures under quizalofop-p-ethyl exposure: samples from the control groups (CKD30, CKD60, CKD90) exhibited a relatively clustered distribution pattern, while those from the treatment groups (QD30, QD60, QD90) were significantly separated from the control groups, with the treatment group samples at different depths showing a scattered distribution.

The relative abundance of soil microorganisms at the phylum level among different treatment groups (Figure 3D) showed that the control groups (CKD series) had relatively rich microbial phyla, with p_*Actinobacteriota* (33.7–50.3%), p_*Proteobacteria* (9.8–22.6%), p_*Chloroflexi* (13–18.7%), and other phyla accounting for a certain proportion. In contrast, in the quizalofop-p-ethyl treatment groups (QD series), the overall species abundance of bacteria decreased, while the abundances of *Proteobacteria* (68–84.7%) and *Firmicutes* (increased from 2–4.7% to 10–20%) increased. Among these, *Proteobacteria* exhibited the most significant change and gradually became the dominant phylum. Particularly in the QD60 and QD90 groups, the proportion of p_*Proteobacteria* reached nearly 84.7%.

At the species level (Figure 3E), quizalofop-p-ethyl induction increased the bacterial diversity in soils at different depths. The species distribution in the treatment groups (QD series) showed obvious specificity: bacterial groups such as *Sphingobium lactosutens DS20*, *Flavobacterium anhuiense*, and *Sinorhizobium meliloti ClAM1775* were enriched in the 0–30 cm soil layer, while groups including *Agrobacterium rubi* and *Methylobacterium extorquens DSM_13060* were increased in the middle and deep soil layers. These changes in species-group associations further confirm the selective effect of quizalofop-p-ethyl on soil microbial communities, leading to the enrichment of specific microbial species as dominant taxa in the treatment groups.

### 3.3 Functional changes of soil bacterial communities induced by quizalofop-p-ethyl

**Figure 4:**
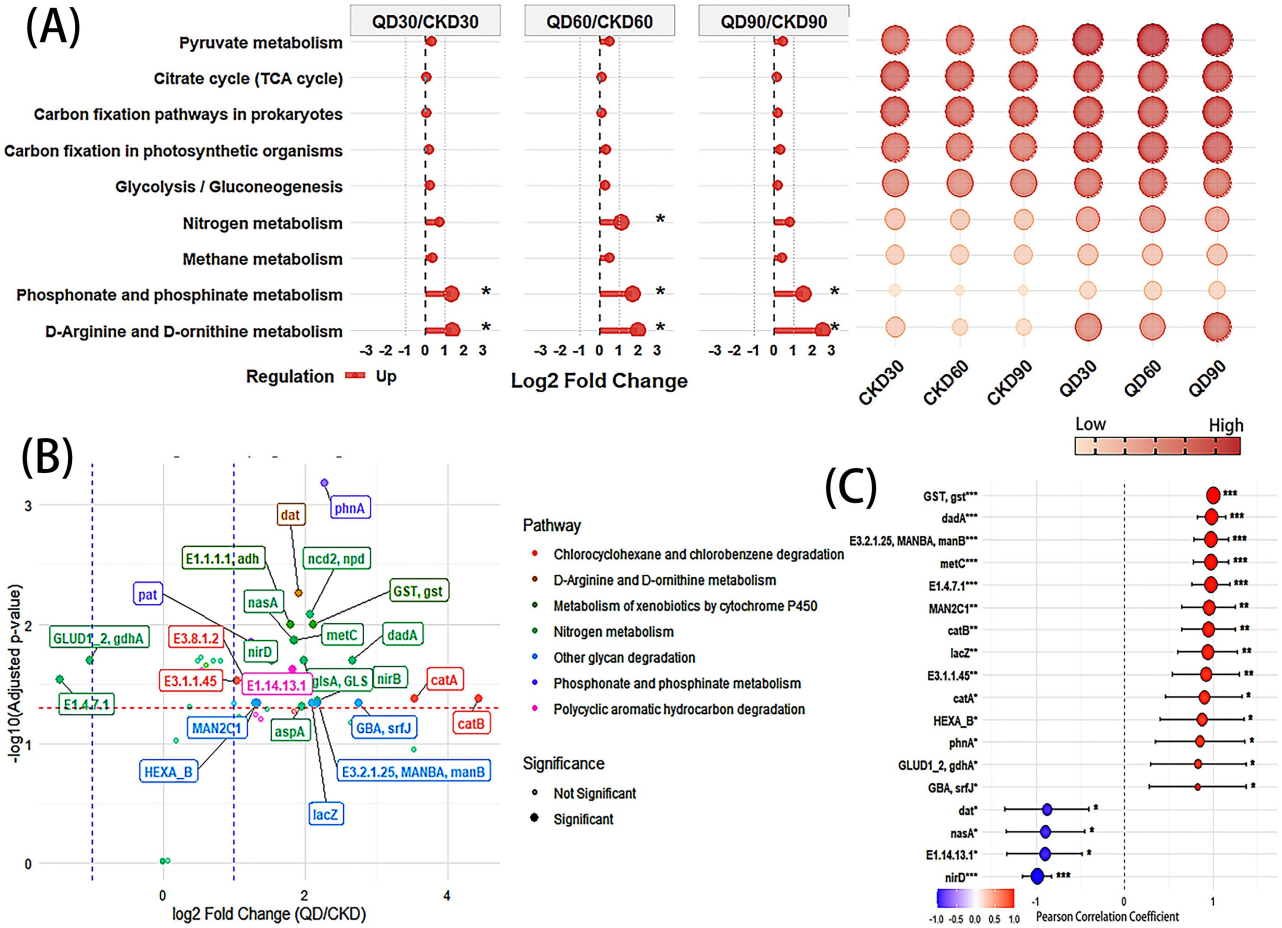
Expression of core functional pathways in soil carbon (C), nitrogen (N), and phosphorus (P) cycles and correlation analysis of differential genes under quizalofop-p-ethyl stress: **(A)** comparison chart of changes in core C, N, and P cycle pathways between treatment groups (QD) and control groups (CKD) in soils at different depths, as well as an expression level bubble chart; **(B)** volcano plot of differentially expressed genes (DEGs) in quizalofop-p-ethyl degradation-related pathways (showing gene upregulation/downregulation trends and significance); **(C)** forest plot of correlations between significantly differentially expressed genes and the relative abundance of *Proteobacteria* (including Pearson correlation coefficients and significance annotations).

PICRUSt functional prediction and correlation analysis systematically revealed the response patterns of functional pathways in soil bacterial communities under quizalofop-p-ethyl stress. We found that functional changes were highly synergistic with community structure remodeling (especially the dominance of the phylum Proteobacteria). Analysis of key pathways involved in soil carbon (C), nitrogen (N), and phosphorus (P) cycles (Figure 4A) showed that in the quizalofop-p-ethyl treatment groups (QD), the expression levels of pathways including pyruvate metabolism, citrate cycle (TCA cycle), carbon fixation pathways in prokaryotes, carbon fixation in photosynthetic organisms, glycolysis/gluconeogenesis, nitrogen metabolism, methane metabolism, phosphonate and phosphinate metabolism, and D-arginine and D-ornithine metabolism were all upregulated compared with the control groups (CKD), with a distinct depth-dependent pattern. Further verification through correlation analysis (Supplementary Figure 1) revealed that the expression levels of the above core C-N-P cycle pathways were significantly positively correlated with the relative abundance of *Proteobacteria* (*r* = 0.68–0.82, *p* < 0.05).

Quizalofop-p-ethyl (C₁₉H₁₇ClN₂O₄) is an aryloxyphenoxypropionate herbicide widely used for controlling gramineous weeds. We found that pathways related to the degradation of the herbicide quizalofop-p-ethyl, such as metabolism of xenobiotics by cytochrome P450, chlorocyclohexane and chlorobenzene degradation, and polycyclic aromatic hydrocarbon degradation, were significantly upregulated (Supplementary Figure 2). Analysis of genes enriched in these significantly upregulated pathways revealed 25 genes with significant changes (Figure 4B). Among them, the *gst* in metabolism of xenobiotics by cytochrome P450; *dadA*, *metC*, *nirD*, *nasA*, *GLUD1_2*, *gdhA*, and E1.4.7.1 genes in nitrogen metabolism; E3.1.1.45, *catB*, and *catA* genes in chlorocyclohexane and chlorobenzene degradation; *lacZ*, *HEXA_B*, E3.2.1.25, *MANBA*, *manB*, *MAN2C1*, *GBA*, and *srfJ* genes in other glycan degradation; the *phnA* gene in phosphonate and phosphinate metabolism; the *dat* gene in D-arginine and D-ornithine metabolism; and the E1.14.13.1 gene in polycyclic aromatic hydrocarbon degradation were all significantly correlated with the abundance of *Proteobacteria* (Figure 4C).

## 4 Discussion

### 4.1 Vertically stratified changes in bacterial community structure and succession mechanism of dominant taxa induced by quizalofop-p-ethyl

Community structure analysis (Figures 3A,B) revealed that the remodeling of soil bacterial community structure in wheat fields induced by quizalofop-p-ethyl exhibited significant vertically stratified characteristics. This phenomenon is directly associated with the synergistic effects of microhabitat heterogeneity along soil depth gradients and the ecological adaptability of bacterial taxa. From the perspective of α- and β-diversity responses, the difference in species richness and community evenness between the quizalofop-p-ethyl treatment groups (QD) and control groups (CKD) expanded significantly with increasing soil depth, particularly in the 60–90 cm deep soil (Figures 3A,B). PCoA analysis showed that samples from the control groups (CKD) exhibited a clustered distribution, while those from the treatment groups (QD) were not only significantly separated from the control groups but also highly scattered among different depths (Figure 3C). These results clearly indicate that the buffering capacity of deep soil bacterial communities against quizalofop-p-ethyl stress is significantly weaker than that of topsoil. The core reason lies in the inherent lack of functional redundancy in deep soil microorganisms. Compared to topsoil, deep soil microorganisms lack sufficient nutritional support to resist stress, making their community structure more susceptible to remodeling. Dominated by oligotrophic taxa, deep soil microbial communities struggle to maintain stability through functional compensation when facing chemical stress (Du et al., 2023). Microhabitat heterogeneity caused by soil depth directly regulates the intensity of microbial responses to disturbances. Due to resource limitations, deep soil microbial communities are significantly more sensitive to pollutants than topsoil (Barbour et al., 2022); studies on the distribution patterns of soil profile microorganisms have confirmed that deep soil microbial diversity has a lower response threshold to chemical stress. Taxa such as *Actinobacteriota* and *Chloroflexi* are more likely to be eliminated, ultimately leading to a decrease in diversity (Liu et al., 2022).

From the perspective of vertical succession in species composition, the depth-dependent dominance of Proteobacteria at the phylum level is the most core characteristic (Figure 3D), and this successional pathway is directly related to the ecological adaptability of taxa. At the species level, specifically enriched bacterial groups emerged in soils at different depths (Figure 3E). In the 0–30 cm topsoil, *Sphingobium* taxa were significantly enriched. This genus is known to promote plant nitrogen uptake and root development (Wang et al., 2025), and its enrichment may help alleviate the interference of herbicides on nitrogen metabolism in topsoil. In contrast, the enrichment of *Agrobacterium rubi* and *Methylobacterium extorquens DSM_13060* in the middle and deep soil layers reflects the selection pressure exerted by the deep-soil environment on bacterial groups with specific metabolic types. Agrobacterium has demonstrated potential for organic matter transformation during solid-state fermentation (Chen et al., 2024), while *Methylobacterium extorquens* may play a key role in deep-soil carbon cycling due to its efficient carbon dioxide fixation capacity (von Borzyskowski et al., 2018), studies on the variation patterns of depth-stratified characteristics of soil microorganisms during alpine meadow restoration (Zhou et al., 2024), the coupling mechanism between wheat yield, soil nutrients, and bacterial communities under exogenous nutrient input (Fan et al., 2025), and the regulatory effects of combined herbicide and safener application on rhizosphere microorganisms (Feng et al., 2025), all confirm that soil microbial structure and function exhibit significant depth dependence.

### 4.2 Vertical response of bacterial community functional pathways and synergistic regulation mechanism of carbon-nitrogen-phosphorus cycles

Under quizalofop-p-ethyl stress, the core pathways of soil carbon (C), nitrogen (N), and phosphorus (P) cycles in wheat fields exhibited significant vertical response characteristics. Notably, the changes in these pathways formed a tight coupling relationship with the depth-dependent dominance of *Proteobacteria* and the differential expression of key genes. Meanwhile, herbicide degradation-related pathways indirectly promoted the C cycle by providing additional carbon sources (Kong et al., 2025), which fully echoes the functional analysis results (Section 3.3).

From the perspective of the overall vertical changes in C-N-P cycle pathways, the expression levels of core pathways in the three cycles were significantly upregulated in the quizalofop-p-ethyl treatment groups (QD), with a more pronounced upregulation trend as soil depth increased (Figure 4A). This phenomenon is consistent with the general pattern of long-term multiple herbicide application affecting soil microbial functions and nutrient cycling (Wen et al., 2025), indicating that herbicide stress may systematically alter the metabolic landscape of soil microorganisms. Further correlation analysis revealed a significant positive correlation between the expression levels of C-N-P cycle-related pathways and the relative abundance of *Proteobacteria* (Supplementary Figure 1), and this synergistic upregulation effect was more prominent in deep soil (Supplementary Figure 2). These results directly demonstrate that Proteobacteria is the core functional taxon driving the vertical upregulation of soil C-N-P cycle pathways under quizalofop-p-ethyl stress. Its depth-dependent dominance in the treatment groups (Figure 3D) provides the necessary functional carriers for the activation of key pathways in soil C-N-P cycling. Multiple studies support the critical role of *Proteobacteria* in maintaining soil element cycling. For example, research has shown that the herbicide diflufenican significantly affects the composition and functions of sugarcane rhizosphere microorganisms during its field degradation, with *Proteobacteria* playing a central role in sustaining carbon and nitrogen cycles (Wang et al., 2023). In farmland soils chronically exposed to nicosulfuron, the bacterial community structure also undergoes significant changes accompanied by adaptive adjustments in nutrient metabolic functions (Ma et al., 2022), further confirming that modifying soil microbial communities (especially enhancing *Proteobacteria*-related functions) can improve C-N-P cycling capacity (Hu et al., 2025). Meanwhile, the role of *Proteobacteria* as a key “functional carrier” (Wu et al., 2022), may be closely related to its diverse phosphorus acquisition strategies and its important position in rhizosphere ecosystems (Wu et al., 2023; Krause et al., 2025).

Under quizalofop-p-ethyl stress, metabolic pathways related to herbicide degradation (Li et al., 2021; Xue et al., 2023; Lavecchia et al., 2024), in the soil bacterial community of wheat fields—including metabolism of xenobiotics by cytochrome P450, chlorocyclohexane and chlorobenzene degradation, and polycyclic aromatic hydrocarbon degradation—were all significantly upregulated, and showed a significant positive correlation with the relative abundance of Proteobacteria (Supplementary Figures 1, 2). This indicates that Proteobacteria may play a key role in herbicide degradation. The application of quizalofop-p-ethyl not only activated pathways associated with xenobiotic metabolism but also significantly enhanced the carbon metabolic activity of soil bacteria. This is reflected by the widespread upregulation of core carbon metabolic pathways (e.g., pyruvate metabolism, citrate cycle, and glycolysis/gluconeogenesis) in the treatment groups (Li et al., 2025; Zhang Y. et al., 2025a), herbicide degradation drives the activation of microbial carbon metabolism: while serving as a potential carbon source, the herbicide may also indirectly regulate carbon cycle functions by altering community structure (Zhang Y. et al., 2025a). A total of 25 significantly differentially expressed genes (DEGs) were identified from 7 significantly upregulated pathways (Figure 4B), among which 18 showed an extremely significant positive correlation with the relative abundance of Proteobacteria. These genes include: *dadA*, *metC*, *nirD*, *nasA*, *gdhA*, and E1.4.7.1 involved in nitrogen metabolism (Ma et al., 2022; Zhang Z. et al., 2025a), *gst*, E3.1.1.45, *catA*, and *catB* associated with xenobiotic degradation (Zhang S. et al., 2025), and *lacZ*, *HEXA_B*, E3.2.1.25, *manB*, *MAN2C1*, *GBA* (*srfJ*), *phnA*, and *dat* involved in polysaccharide degradation and phosphonate metabolism. The synergistic upregulation of these functional genes not only enhances the degradation capacity of bacteria toward quizalofop-p-ethyl but also reflects that under herbicide stress, soil microorganisms achieve the coupling of detoxification and energy metabolism through metabolic network remodeling. This further promotes the rebalancing of key element cycles (e.g., carbon, nitrogen, and phosphorus) at the community and ecosystem levels (Ma et al., 2025).

### 4.3 Scientific application insights for quizalofop-p-ethyl based on community and functional responses

Based on the vertical impact patterns of quizalofop-p-ethyl on bacterial community structure, degradation functions, and carbon (C)-nitrogen (N)-phosphorus (P) cycles revealed in this study, targeted management directions can be provided for the scientific application of quizalofop-p-ethyl in wheat fields. From the perspective of soil depth, deep soil bacterial communities are more sensitive to quizalofop-p-ethyl, with slower degradation rates and a higher risk of herbicide accumulation. Therefore, it is necessary to reduce the downward migration of the herbicide to deep soil layers in practical application. Given the core role of *Proteobacteria* in community structure dominance, degradation function activation, and C-N-P cycle driving, soil environments can be regulated in actual production to promote the growth of this phylum. This will further enhance the soil’s self-purification capacity for quizalofop-p-ethyl and material cycling efficiency, thereby avoiding soil fertility decline caused by excessive simplification of community structure or inhibition of functional pathways.

## 5 Conclusion

This study systematically revealed the vertical differentiation patterns of soil bacterial communities and their ecological functional response mechanisms in wheat fields under quizalofop-p-ethyl stress. The results indicated that quizalofop-p-ethyl treatment significantly induced depth-dependent changes in soil bacterial community structure. Particularly in deep soil, the phylum *Proteobacteria* exhibited obvious dominant enrichment and became the core taxon driving the upregulation of carbon (C), nitrogen (N), and phosphorus (P) cycle functions. This study identified the “initial response threshold” of soil microorganisms to quizalofop-p-ethyl (approximately 50 mg L^−1^), and this threshold showed higher ecological sensitivity in deep soil. Functional analysis further demonstrated that the expression levels of key pathways related to C, N, and P metabolism were all significantly positively correlated with the relative abundance of *Proteobacteria*, with N and P cycle-related pathways being significantly enhanced with increasing soil depth. In addition, quizalofop-p-ethyl degradation-related pathways and their enriched functional genes were also closely associated with *Proteobacteria*. By providing additional carbon sources for microbial metabolism during herbicide degradation, these pathways synergistically enhanced soil carbon fixation and C cycle capacity. The depth-dependent dominance of *Proteobacteria* under quizalofop-p-ethyl stress is not only a key characteristic of microbial community structural response but also undertakes the dual functions of “pollutant degradation” and “C-N-P cycle driving.” Its deep enrichment represents a critical manifestation of soil microorganisms achieving metabolic network remodeling and ecological function rebalancing under chemical stress.

## Data Availability

The data presented in the study are deposited in the NCBI repository (https://www.ncbi.nlm.nih.gov), accession number PRJNA1431111.
