# Supplementary material for: Deep enrichment of soil Proteobacteria and its coupled response to carbon, nitrogen, and phosphorus cycles under quizalofop-p-ethyl stress
Source: Front Microbiol. 2026 Mar 12;17:1766973. doi: 10.3389/fmicb.2026.1766973 (PMC13017852; doi:10.3389/fmicb.2026.1766973)
Supplement: Supplementary file 1 [file Supplementary_file_1.docx]

**Supplementary** **Table 1** Information of soil sample villages

| Village Name | Code | Latitude and Longitude |
| --- | --- | --- |
| Fengrun Village, Xiaohaizi Town | A1 | 41°47′N，113°71′E |
| Xiejiafang, Sandaqing Township | A2 | 41°52′N，113°46′E |
| Haiyan Village, Sandaqing Township | A3 | 41°44′N，113°52′E |
| Dachengzi Village, Qitai Town | A4 | 41°54′N，113°58′E |
| Botun Line, Boli Village, Boli Township | A5 | 41°76′N，113°71′E |
| Yangchang Village, Tunkendui Town | A6 | 41°75′N，113°46′E |
| Zhangguo, Xiaojingzi, Dakulian Township, Xinghe County | A7 | 41°25′N，113°70′E |
| Lijun, Dakulian Township, Xinghe County | A8 | 41°22′N，113°77′E |
| Three-Year Continuous Cropping, Daliuhao Village, Houqi Banner | B1 | 41°22′N，113°29′E |
| Near Dahulun Town Middle School, Zhangbei County | B2 | 41°33′N，115°21′E |
| Yuanshanzi, Fengzhen City | B3 | 40°53′N，113°47′E |
| Heigeda Township, Fengzhen City | B4 | 40°41′N，113°32′E |
| Houbuddi, Gonggouyan, Liusumu Township, Liangcheng County | B5 | 40°45′N，112°52′E |
| Gaojiadi, Houqi Banner | B6 | 41°43′N，113°17′E |
| Chahaying, Sanchakou, Houqi Banner | B7 | 40°96′N，112°95′E |
| Shibatai, Zhuozishan City | B8 | 40°92′N，112°91′E |
| Hezigou Brigade, Dayushu, Zhuozishan City | C1 | 40°91′N，112°56′E |
| Jincheng Agricultural Cooperative, Xin'anying, Meiguiying Town, Qianqi Banner | C2 | 41°03′N，113°34′E |
| Pangjiacun, Meiguiying Town, Qianqi Banner | C3 | 41°01′N，113°35′E |
| Li Jinlong's Farm, Qianqi Banner | C4 | 41°02′N，109°13′E |
| Bayin Qiganliang, Qianqi Banner | C5 | 40°97′N，113°34′E |
| Nanying, Qianqi Banner | C6 | 41°05′N，113°21′E |
| Zhang Lanying, Qianmani Tu Village, Baolongshan Town, Horqin Left Middle Banner | C7 | 44°00′N，122°77′E |
| Tian Weidong, Tian Baotun, Jiamatu Town, Horqin Left Middle Banner | C8 | 44°03′N，122°98′E |
| Shixing Village, Jiamatu Town, Horqin Left Middle Banner | D1 | 44°10′N，122°93′E |
| Lianhe Tun, Jiamatu Town, Horqin Left Middle Banner | D2 | 44°10′N，122°91′E |
| Lianmin Zhutun, Jiamatu Town, Horqin Left Middle Banner | D3 | 44°04′N，122°04′E |
| Harigan Tu Village, Yaolin Maodu Town, Horqin Left Middle Banner | D4 | 44°15′N，122°52′E |
| Nantaolin Village, Yaolin Maodu Town, Horqin Left Middle Banner | D5 | 44°10′N，122°34′E |
| Minzhu Village, Shebotu Town, Horqin Left Middle Banner | D6 | 44°04′N，122°04′E |
| Haihe Tun Village, Xibohua Town, Horqin Left Middle Banner | D7 | 44°00′N，121°63′E |
| Guyushu Village, Xiaojiejia Town, Kailu County | D8 | 43°85′N，121°55′E |

**Supplementary Table 2** Main reagents

| Reagent | Company |
| --- | --- |
| Acetone | Sinopharm Chemical Reagent Co., Ltd. |
| K₂HPO₄ | Sinopharm Chemical Reagent Co., Ltd. |
| KH₂PO₄ | Sinopharm Chemical Reagent Co., Ltd. |
| MgSO₄·7H₂O | Sinopharm Chemical Reagent Co., Ltd. |
| NaCl | Sinopharm Chemical Reagent Co., Ltd. |
| Agar Powder | Sinopharm Chemical Reagent Co., Ltd. |
| Tryptone | Sinopharm Chemical Reagent Co., Ltd. |
| Yeast Extract Powder | Sinopharm Chemical Reagent Co., Ltd. |

**Supplementary Table 3** Main equipment

| Instrument | Model | Manufacturer |
| --- | --- | --- |
| High-Pressure Steam Sterilizer | MLS-3020 | Sanyo Electric Co., Ltd. (Japan) |
| Shaking Incubator | HZQ-F160 | Harbin Donglian Electronics Co., Ltd. |
| High-Speed Refrigerated Centrifuge | CF16RXII | Hitachi, Ltd. (Japan) |
| Vortex Mixer | VORTEX-5 | Haimen Qilinbeier Instrument Manufacturing Co., Ltd. |
| Electronic Analytical Balance | AB104-N | Mettler-Toledo Instruments Co., Ltd. |
| Clean Bench | DL-CJ-2N | Beijing Donglian Ha'er Instrument Manufacturing Co., Ltd. |
| Pipette | BG-easy PIPET | Beijing Bio-Gene Technology Co., Ltd. |
| Electrothermal Constant-Temperature Incubator | DNP-9162 | Shanghai Jinghong Experimental Equipment Co., Ltd. |

**Supplementary Table 4** dilution ratio

| Final Concentration (mg/L) | 50 | 100 | 150 | 200 | 250 | 300 |
| --- | --- | --- | --- | --- | --- | --- |
| Stock Solution Volume (mL) | 0.2 | 0.4 | 0.6 | 0.8 | 1.0 | 1.2 |
| Medium Volume (mL) | 99.9 | 99.8 | 99.7 | 99.6 | 99.5 | 99.4 |


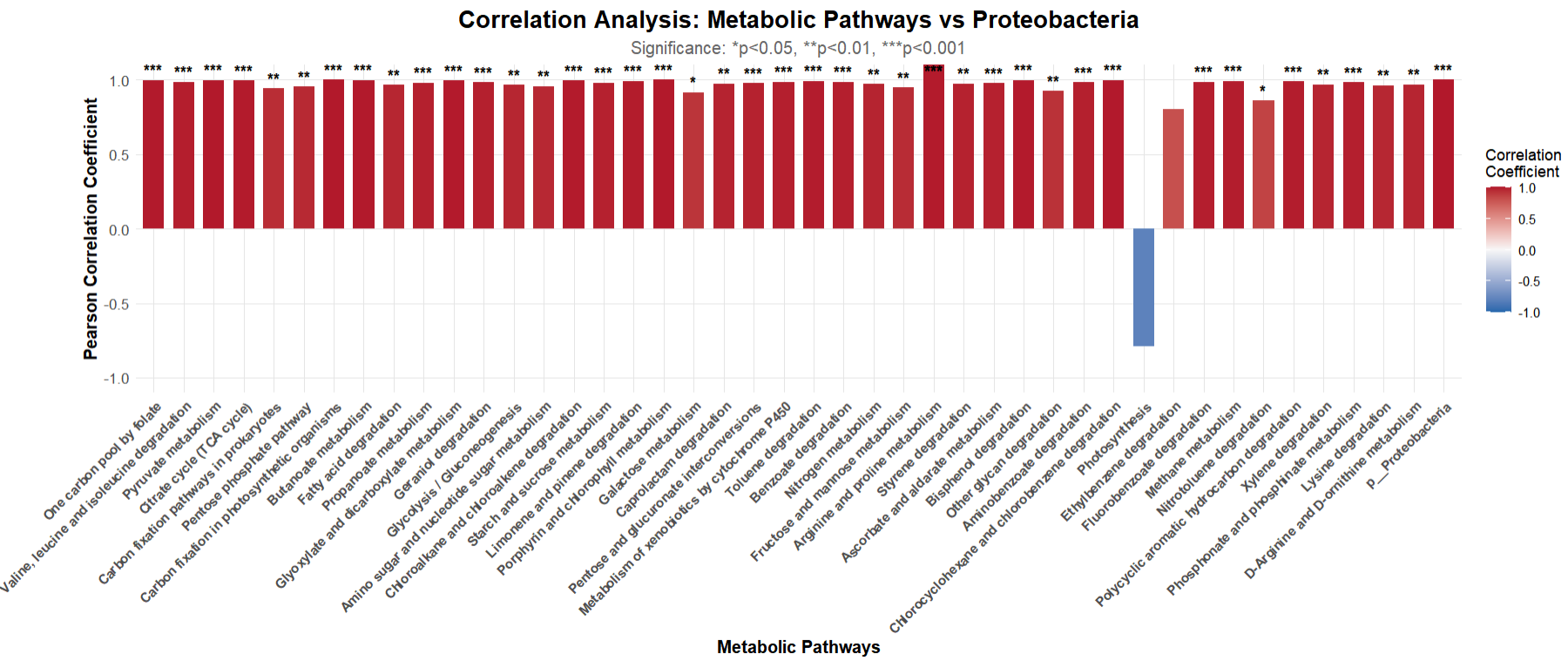


**Supplementary Figure 1**. Changes in carbon, nitrogen, and phosphorus cycle-related pathways and herbicide degradation-related pathways, and their correlation with the abundance of *Proteobacteria.*
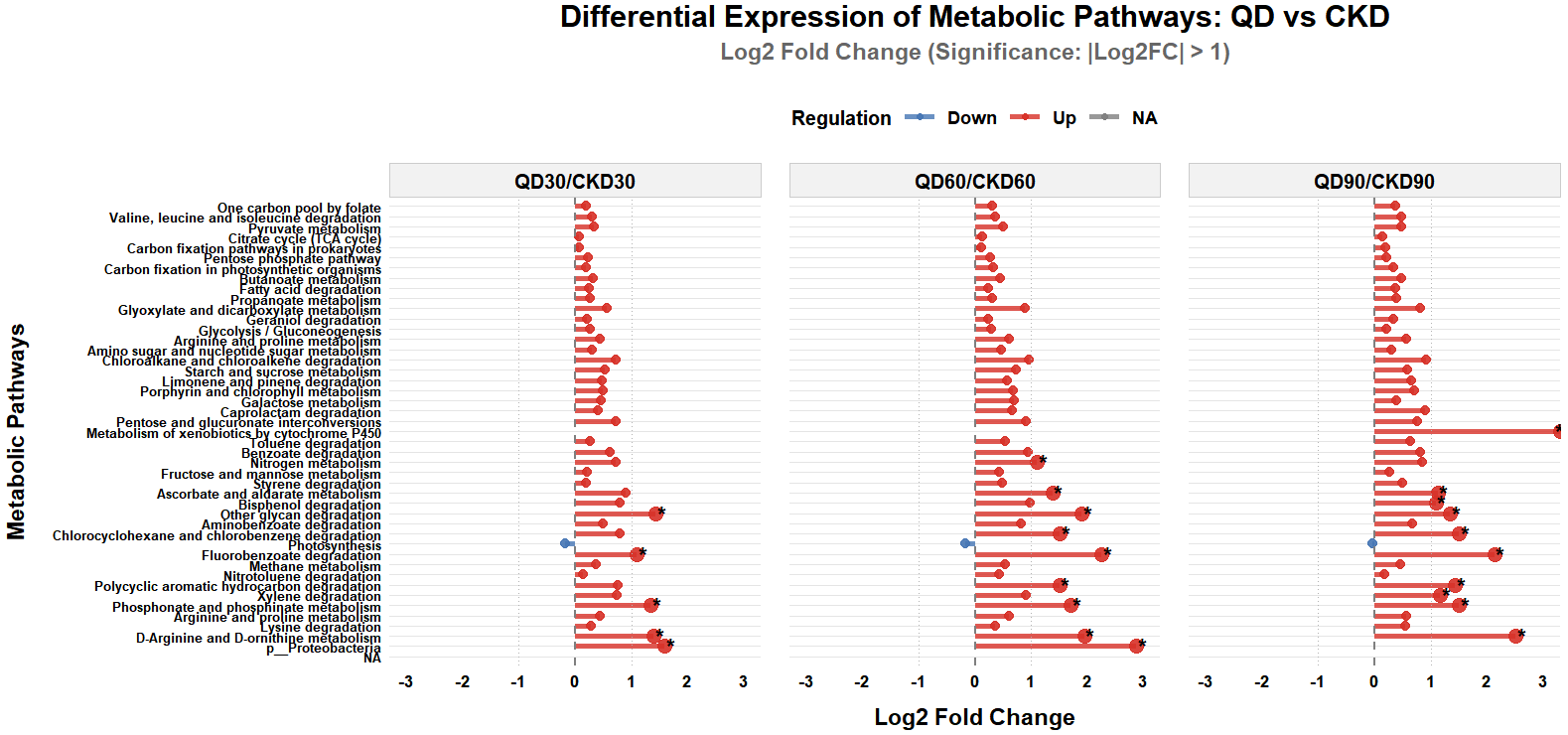


**Supplementary Figure 2**. Upregulated and downregulated changes of carbon, nitrogen, and phosphorus cycle-related pathways and herbicide degradation-related pathways at different depths (0-30 cm, 30-60 cm, 60-90 cm) under herbicide treatment compared with the control group.
